# Targeted DNA Sequencing Reveals Distinct Rare-Variant Profiles in Pulmonary Hypertension Associated with Chronic Lung Diseases

**DOI:** 10.3390/ijms27208992

**Published:** 2026-10-10

**Authors:** Adelaida Bosacoma, Agustín R. Garcia, Irene Madrigal, Juan-José Lozano, Julia Sidorova, Daniel Aguilar, Isabel Blanco, Ylenia Roger, Olga Tura-Ceide, Anna Sardiné-Rama, Clara Martín-Ontiyuelo, Diego A. Rodríguez-Chiaradía, Manuel López-Meseguer, Fernanda Hernandez-Gonzalez, Jesús Ribas, Xavier Pomares, Salud Santos, María Molina-Molina, Rita Fernández-Hernández, Victor I. Peinado, Joan A. Barberà

**Affiliations:** 1 Department of Pulmonary Medicine, Hospital Clinic-IDIBAPS, University of Barcelona, 08036 Barcelona, Spain; bosacoma@recerca.clinic.cat (A.B.); agustin.garciaroca@gmail.com (A.R.G.); iblanco2@clinic.cat (I.B.); yleniaroger@gmail.com (Y.R.); olgaturac@gmail.com (O.T.-C.); sardine@recerca.clinic.cat (A.S.-R.); clmartin@clinic.cat (C.M.-O.); fhernandez@clinic.cat (F.H.-G.); rifernandez@recerca.clinic.cat (R.F.-H.); vpeinado@recerca.clinic.cat (V.I.P.); 2 Biomedical Research Networking Centre in Respiratory Diseases (CIBERES), 28029 Madrid, Spain; darodriguez@psmar.cat (D.A.R.-C.); manuel.lopez@vallhebron.cat (M.L.-M.); saludsantos@bellvitgehospital.cat (S.S.); mariamolinamolina@hotmail.com (M.M.-M.); 3 Biochemistry and Molecular Genetics Department, Hospital Clinic of Barcelona and Fundació de Recerca Clínic Barcelona-Institut d’Investigacions Biomediques August Pi i Sunyer (FRCB-IDIBAPS), 08036 Barcelona, Spain; imadbajo@clinic.cat; 4 Bioinformatics Platform, Biomedical Research Networking Centre in Hepatic and Digestive Disease (CIBEREHD), 28029 Madrid, Spain; juanjo.lozano@ciberehd.org (J.-J.L.); julia.a.sidorova@gmail.com (J.S.); 5 Vall d’Hebron Institute of Oncology (VHIO), Vall d’Hebron Barcelona Hospital Campus, 08035 Barcelona, Spain; daniel.aguilar.mail@protonmail.com; 6 ERN-LUNG (European Reference Network on Rare Respiratory Diseases), Villarroel 170, 08036 Barcelona, Spain; 7 Translational Research Group on Cardiovascular Respiratory Diseases (CAREs), Dr. Josep Trueta University Hospital de Girona, Avinguda de França, 17007 Girona, Spain; 8 Girona Biomedical Research Institute (IDIBGI-CERCA), Santa Caterina Hospital de Salt, 17190 Salt, Spain; 9 Department of Pulmonary Medicine, Hospital del Mar Research Institute, Department of Medicine and Life Sciences (MELIS), Universitat Pompeu Fabra (UPF), 08003 Barcelona, Spain; 10 Department of Pulmonary Medicine, Hospital Vall d’Hebron, 08035 Barcelona, Spain; 11 Department of Pulmonary Medicine, Hospital Universitari de Bellvitge, IDIBELL, University of Barcelona, Hospitalet de Llobregat, 08907 Barcelona, Spain; jribass@bellvitgehospital.cat; 12 Department of Pulmonary Medicine, Parc Taulí Hospital Universitari, Institut d’Investigació i Innovació Parc Taulí (I3PT-CERCA). Universitat Autònoma de Barcelona, 08208 Sabadell, Spain; jpomares@tauli.cat; 13 Department of Experimental Pathology, Institut d’Investigacions Biomèdiques de Barcelona (IIBB), CSIC-IDIBAPS, 08036 Barcelona, Spain

**Keywords:** pulmonary hypertension, chronic obstructive pulmonary disease, interstitial lung disease, idiopathic pulmonary arterial hypertension, variants, *BMPR2*

## Abstract

The mechanisms underlying the development and severity of pulmonary hypertension (PH) in chronic obstructive pulmonary disease (COPD) and interstitial lung disease (ILD) remain poorly understood. To investigate the contribution of genetic factors, we performed targeted sequencing of PH-related genes in 63 patients with COPD, 51 with ILD, and 38 with idiopathic pulmonary arterial hypertension (IPAH), stratifying COPD and ILD patients according to PH severity, assessed by right heart catheterisation. Patients with ILD-associated PH harboured fewer genetic variants than those without PH, whereas an opposite trend was observed in COPD-associated PH. Variants of uncertain significance (VUS) in PAH-associated genes (*BMPR2*, *BMPR1B*) were identified in two out of 28 patients with severe COPD-PH and in one with nonsevere ILD-PH (*SMAD9*). VUS in extracellular matrix-related genes (*THBS1*, *LOXL2*) were also identified in four patients with severe COPD-PH, and in inflammatory-related genes *(TNFAIP6*) in two patients with ILD-PH. Pathogenic/likely pathogenic variants in PAH-associated genes were exclusive to IPAH. We conclude that there is limited genetic overlap between COPD-PH and ILD-PH, with a stronger genetic contribution to PH in COPD, particularly in its severe form, where a subset of patients carries VUS in PAH-associated genes or genes involved in vascular remodelling.

## 1. Introduction

Pulmonary hypertension (PH) is common in patients with chronic lung disease (CLD), particularly chronic obstructive pulmonary disease (COPD) and interstitial lung disease (ILD). It is associated with poor survival and reduced exercise capacity [1,2]. A subset of patients with CLD develop severe PH, which is associated with even poorer survival [3]. Given the high prevalence of CLD, there is ongoing debate about whether severe CLD-PH represents advanced pulmonary vascular disease in a patient with CLD or the concomitant presence of idiopathic pulmonary arterial hypertension (IPAH) in a patient with underlying CLD [4]. Despite the significant burden and clinical relevance of PH in CLD [1], the mechanisms underlying its development and severity remain poorly understood.

Assessment of genetic variants is a powerful approach for identifying biomarkers related to disease onset, progression, and prognosis. Genome-wide association studies (GWASs) have identified loci associated with susceptibility to chronic obstructive pulmonary disease (COPD) and interstitial lung disease (ILD) [5]. In PH, most genetic studies have focused on heritable and idiopathic pulmonary arterial hypertension (PAH). Genes with definitive evidence supporting their role in causing the disease include: *BMPR2*, *ACVRL1*, *ATP13A3*, *CAV1*, *EIF2AK4*, *ENG*, *GDF2*, *KCNK3*, *KDR*, *SMAD9*, *SOX17* and *TBX4.* Nine other genes have limited or disputed evidence of a gene–disease relationship [6].

While the genetic determinants in heritable and idiopathic PAH have been extensively characterised, much less has been studied or is known about the potential genetic contributors to associated PAH subtypes [7] or to other PH groups. A considerable knowledge gap remains regarding the genetic factors that may underlie the development of PH in patients with COPD or ILD, and it is unclear whether the presence of specific variants increases the risk of PH development in these populations.

Identifying genetic variants and understanding their functional consequences in patients with CLD-associated PH may help elucidate the mechanisms underlying the development of PH in this condition and why a subset of patients develop severe PH. Accordingly, this study investigated whether the presence and severity of PH in COPD and ILD are associated with specific genetic variants. Additionally, we evaluated whether genetic variants identified in COPD- or ILD-associated PH overlap with those observed in IPAH. To this end, we performed targeted sequencing of genes involved in PH-related biological pathways using a customised panel in patients with COPD and ILD, with and without PH, and in patients with sporadic IPAH as a reference group with established genetic associations.

## 2. Results

### 2.1. Patient Characteristics

Patient characteristics are shown in Table 1. Patients with COPD and ILD were older and had a higher proportion of men than those with IPAH. Patients with COPD and severe PH had numerically higher FEV_1_ values, albeit lower diffusing capacity for carbon monoxide (DLCO) and greater hypoxemia than those without PH or with nonsevere PH.

### 2.2. Variant Burden in Chronic Lung Disease-Associated PH

**Figure 1:**
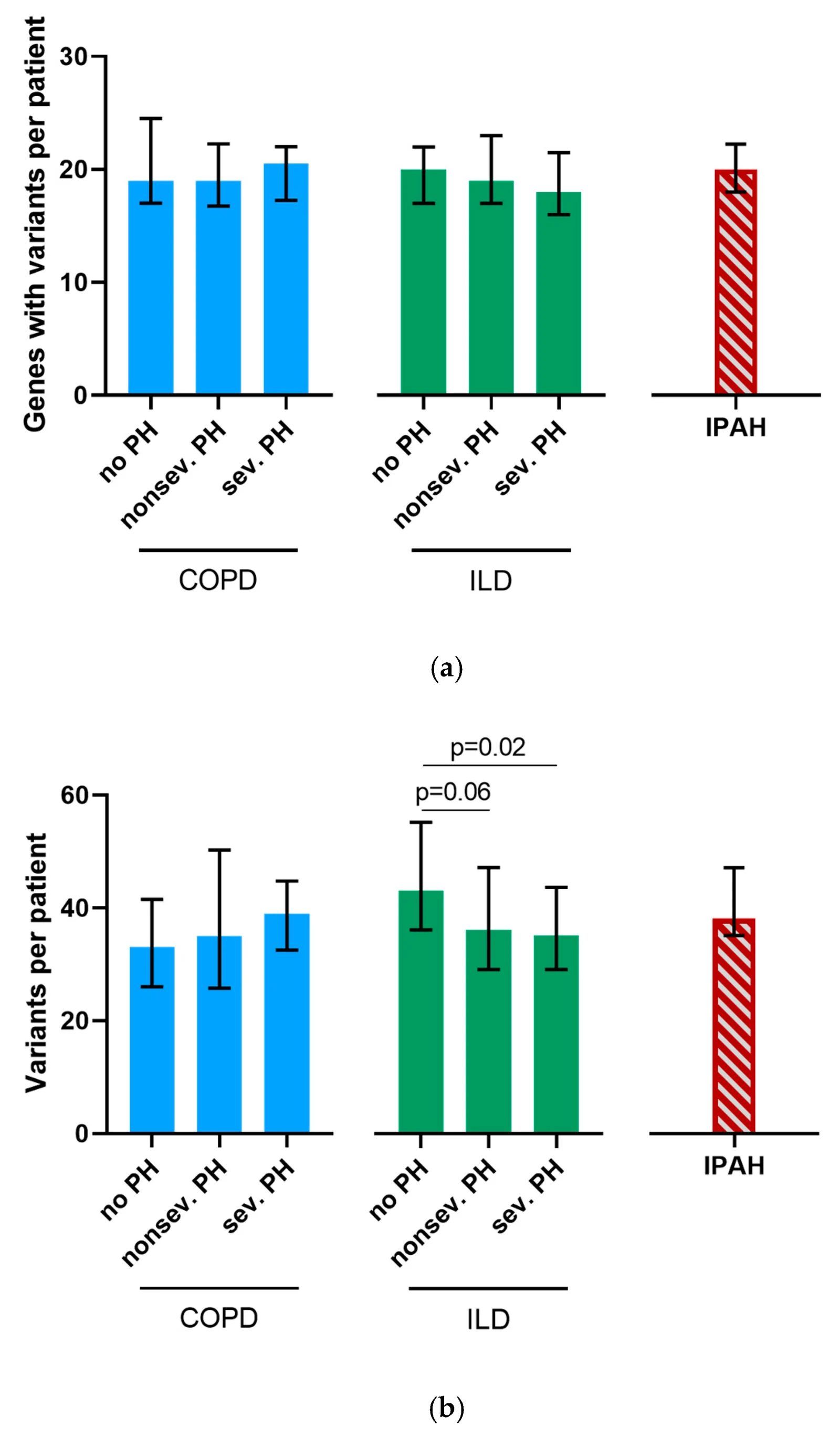
Genetic variant burden in chronic lung disease-associated pulmonary hypertension. (**a**) Number of genes with variants per patient across disease groups and (**b**) number of variants per patient in chronic obstructive pulmonary disease (COPD) and interstitial lung disease (ILD), stratified by hemodynamic severity, and idiopathic pulmonary arterial hypertension (IPAH). Data are presented as medians with interquartile ranges, and *p*-values were calculated using the Kruskal–Wallis test followed by Dunn’s post hoc test. No PH: without pulmonary hypertension (PH); nonsev: nonsevere PH; sev: severe PH.

Among the 150 sequenced genes, all but five harboured variants in at least one patient of the cohort. On average, each patient carried variants in 20 genes, with no significant differences across disease groups or hemodynamic status (Figure 1a). Coverage and callable fraction of all genes did not differ between groups (Supplementary Table S3). Patients with COPD-PH showed a numerically higher number of variants per patient than those without PH (37.5 (IQR 29,47) and 33 (26,41), respectively; *p* = 0.156). In contrast, patients with ILD-PH had significantly fewer variants than those without PH (35.5 (29,45) and 43 (36,55), respectively; *p* = 0.016), particularly among patients with severe PH (Figure 1b).

**Figure 2:**
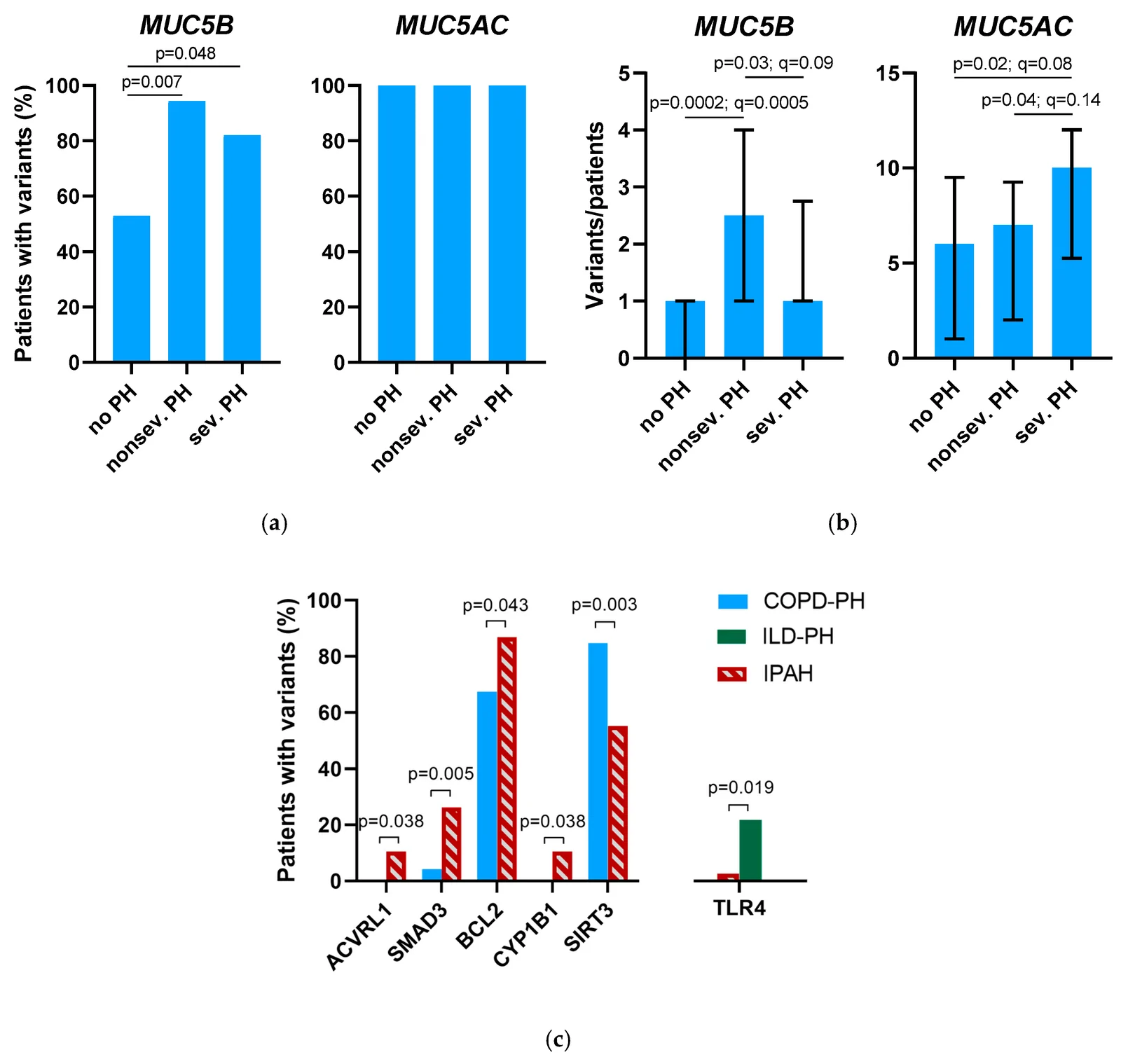
Variant differences between pulmonary hypertension (PH) groups. Panels a and b show variant burden of mucin-5-associated genes in chronic obstructive pulmonary disease (COPD). (**a**) Percentage of patients carrying variants and (**b**) number of gene variants per patient in *MUC5B* and *MUC5AC*. Patients were stratified into no PH, nonsevere (nonsev) PH and severe (sev) PH. (**c**) Percentage of patients with COPD-associated PH (COPD-PH), interstitial lung disease-associated PH (ILD-PH) and idiopathic pulmonary arterial hypertension (IPAH) carrying variants. Data are presented as percentages for (**a**,**c**) and medians with interquartile ranges for (**b**). Group comparisons were performed using Fisher’s exact test for panel (**a**,**b**) and the Kruskal–Wallis test followed by Dunn’s post hoc test (*p*-value for the uncorrected and q-value for the corrected) for panel (**b**). *MUC5B*: mucin-5B; *MUC5AC*: mucin-5AC; *ACVRL1*: activin A receptor-like type-1; *SMAD3*: SMAD family member-3; *BCL2*: B-cell CLL/lymphoma 2; *CYP1B1*: cytochrome P450 family-1 subfamily-B member-1; *SIRT3*: sirtuin-3; *TLR4*: Toll-like receptor-4.

Among the 150 genes analysed, variant burden differences associated with hemodynamic status were confined to mucin-5B (*MUC5B*) and mucin-5AC (*MUC5AC*) in COPD, whereas no PH-related associations were identified in the ILD subgroups. A higher proportion of patients with COPD-PH harboured at least one variant in *MUC5B* compared with patients without PH, particularly those with nonsevere PH, who also exhibited a greater number of variants per patient (Figure 2). In contrast, although all patients with COPD harboured variants in *MUC5AC*, those with severe PH exhibited a higher number of variants per patient than the other subgroups (Figure 2b).

#### Comparison with Idiopathic PAH

Several genes showed differences in variant frequencies between COPD- and ILD-associated PH and IPAH. Compared with COPD-PH, variants in activin A receptor-like type-1 (*ACVRL1*), SMAD family member-3 (*SMAD3*), B-cell CLL/lymphoma 2 (*BCL2*), and cytochrome P450 family-1 subfamily-B member-1 (*CYP1B1*) were more frequent in IPAH, whereas variants in sirtuin-3 (*SIRT3*) were more common in COPD-PH. In contrast, in ILD-PH, the only difference was a higher frequency of Toll-like receptor-4 (*TLR4*) variants compared with IPAH (Figure 2c).

### 2.3. Variants Identified in Chronic Lung Disease-Associated PH and Idiopathic PAH Patients

#### 2.3.1. PAH-Associated Genes

No pathogenic sequence variants were identified in any of the seven genes in the panel with a definitive association with PAH [8] among patients with COPD- or ILD-associated PH, whereas among patients with sporadic IPAH, three carried pathogenic variants in bone morphogenetic protein receptor type-2 (*BMPR2*) and one carried a likely pathogenic variant in *ACVRL1*.

**Figure 3:**
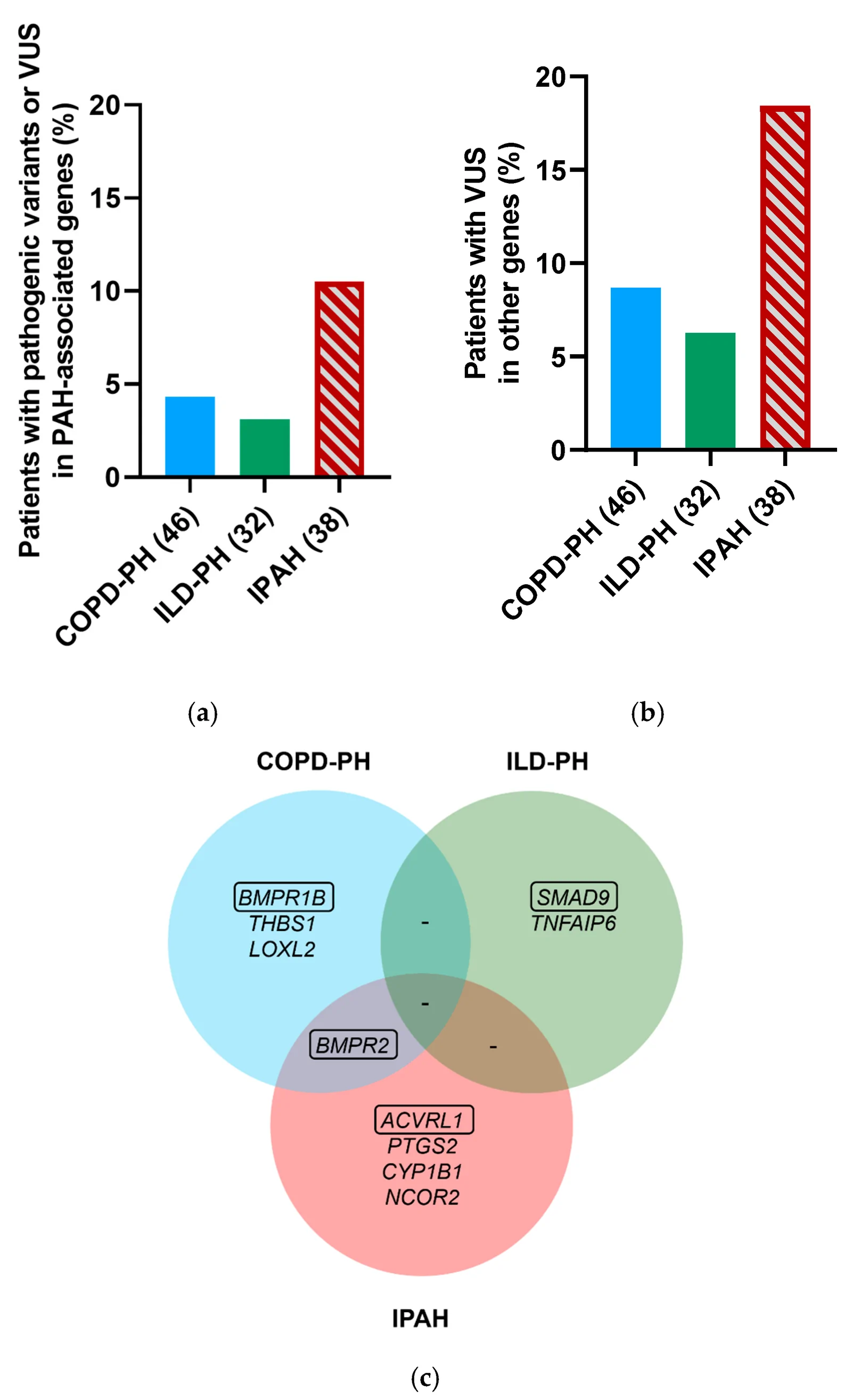
Pathogenic and likely pathogenic variants and variants of uncertain significance (VUS) in chronic lung disease-associated pulmonary hypertension (PH) and idiopathic pulmonary arterial hypertension (IPAH). Percentage of patients with chronic obstructive pulmonary disease-associated PH (COPD-PH), interstitial lung disease-associated PH (ILD-PH) and IPAH carrying (**a**) pathogenic variants or VUS in genes associated with pulmonary arterial hypertension (PAH), and (**b**) VUS in genes not currently associated with PAH. (**c**) Venn diagram illustrating the distribution of pathogenic and likely pathogenic variants and VUS among patients with COPD-PH, ILD-PH and IPAH. Framed genes indicate those with evidence of association with PAH.

Variants of uncertain significance (VUS) in genes definitively associated with PAH were identified in *BMPR2* in one patient with severe COPD-PH and in SMAD family member-9 (*SMAD9*) in one patient with nonsevere ILD-PH (Figure 3a,c, Table 2 and Table 3). Among genes with disputed PAH association, an extremely rare VUS (allele frequency (AF) = 4 × 10^−6^) in bone morphogenetic protein receptor type-1B (*BMPR1B*) was identified in one patient with severe COPD-PH (Table 2 and Table 3).

#### 2.3.2. Genes with No Established Association to PAH

VUS in genes without a described PAH association [8] that were present in two or more patients with PH and absent in those without PH were observed in four of 46 patients with COPD (8.7%) (all of whom had severe PH), in two of 32 with ILD-PH (6.2%), and in seven of 38 with IPAH (18.4%) (Figure 3b,c). These VUS were identified in thrombospondin-1 (*THBS1*), lysyl oxidase-like 2 (*LOXL2*), prostaglandin-endoperoxide synthase-2 (*PTGS2*), TNF-alpha-induced protein 6 (*TNFAIP6*), *CYP1B1*, and nuclear repressor co-receptor 2 (*NCOR2*) genes. All but one were rare variants, defined by an AF <0.01 (Table 2 and Table 3).

**Table 3:** In silico predictions for pathogenic, likely pathogenic variants, and VUS identified in patients with pulmonary hypertension ^1^.

Patient Group	Sex	Age	mPAP (mmHg)	PVR (WU)	Gene	# Uploaded Variation	Chromosome Location	Mane Select	Consequence	Allele Change	Amino Acid Change	ClinVar/ Mobidetails	CADD	Allele Frequency	Revel	Alpha Missense
Variants in PAH-associated genes																
IPAH	F	46	57	18.0	*BMPR2*	rs1085307203	Chr2:203332274	NM_001204.7	Missense	c.280T>C	p.(Cys94Arg)	P	28.9	-	0.953	0.9962
IPAH	F	26	42	16.8	*BMPR2*	rs137852744	Chr2:203395589	NM_001204.7	Missense	c.1040G>A	p.(Cys347Tyr)	P	31	-	0.939	0.9936
IPAH	M	18	76	17.5	*BMPR2*	rs137852748	Chr2: 203421005	NM_001204.7	Stop gained	c.2617C>T	p.(Arg873 *)	P	42	-	-	-
COPD-SPH	M	70	42	8.3	*BMPR2*	rs573463511	Chr2: 203329535	NM_001204.7	Missense	c.80C>T	p.(Ser27Leu)	B/VUS	22.8	1.9 × 10^−5^	0.311	0.1552
COPD-SPH	M	66	35	5.5	BMPR1B *	rs1268550390	Chr4: 96052366	NM_001203.3	Missense, splice region	c.779G>T	p.(Gly260Val)	VUS	34	4 × 10^−6^	0.888	0.9286
IPAH	M	70	49	12.6	*ACVRL1*	rs1234976168	Chr12: 52309927	NM_000020.3	Missense	c.1156C>T	p.(Arg386Cys)	LP	29.9	-	0.788	0.1807
ILD-NSPH	M	78	26	3.7	*SMAD9*	rs941764904	Chr13: 37453823	NM_001127217.3	Missense	c.4C>G	p.(His2Asp)	VUS	23.6	4 × 10^−6^	0.524	0.1683
Variants in other genes																
2 patients with COPD-SPH					*THBS1*	rs377693076	Chr15: 39882031	NM_003246.4	Missense	c.1952C>T	p.(Thr651Met)	B/VUS	22.3	5.4 × 10^−4^	0.229	0.0764
3 patients with IPAH					*CYP1B1*	rs79204362	Chr2: 38298394	NM_000104.4	Missense	c.1103G>T	p.(Arg368Leu)	B/VUS	30	3.1 × 10^−3^	-	0.2003
2 patients with COPD-SPH					*LOXL2*	rs759510676	Chr8: 23167289	NM_002318.3	Missense	c.1772C>A	p.(Thr591Lys)	VUS	22.5	1.7 × 10^−5^	0.086	0.0907
2 patients with IPAH					*NCOR2*	rs200297509	Chr12: 124835279	NM_006312.6	Missense	c.3698G>A	p.(Thr1233Met)	LB/VUS	27.9	8.6 × 10^−4^	0.605	0.6539
2 patients with IPAH					*PTGS2*	rs5273	Chr1: 186643768	NM_000963.4	Missense	c.1532T>G	p.(Val511Ala)	B/VUS	20.4	4.4 × 10^−3^	0.163	0.2664
2 patients with ILD-PH (1 NSPH, 1 SPH)					*TNFAIP6*	rs75961064	Chr2: 152220510	NM_007115.4	Missense	c.148A>G	p.(Thr50Ala)	B/VUS	26.2	1.5 × 10^−2^	0.515	0.4524

^1^ Variants absent in patients without pulmonary hypertension. For each variant, the gene, variant ID (**#**), chromosomal coordinate, cDNA position, predicted consequence, amino acid change, and pathogenicity predictions from CADD, allele frequency, REVEL (Rare Exome Variant Ensemble Learner) and AlphaMissense are provided. Only variants with a CADD Phred score >20 are included. * All genes have definitive evidence except for *BMPR1B*, with disputed evidence [8]. Abbreviations: mPAP: mean pulmonary artery pressure; PVR: pulmonary vascular resistance; WU: Wood units; SPH: severe pulmonary hypertension; NSPH: nonsevere pulmonary hypertension; F: female; M: male; CADD: Combined Annotation-Dependent Depletion; REVEL: Rare Exome Variant Ensemble Learner; P: pathogenic; LP: likely pathogenic; B: benign; VUS: variant of uncertain significance.

## 3. Discussion

In this study, we analysed the genetic variant profiling of patients with CLD-associated PH using a targeted panel of genes involved in PH pathobiology. Patients with COPD-PH exhibited a trend toward greater genetic variability than those without PH, whereas patients with ILD-PH carried significantly fewer variants. No pathogenic sequence variants were identified in either COPD- or ILD-PH. However, a subset of patients harboured VUS in PAH-associated genes, particularly those with COPD-PH. Notably, VUS in COPD-PH were restricted to patients with severe PH, whereas in ILD they were unrelated to hemodynamic severity. Furthermore, differences in variant profiles were observed among COPD-PH, ILD-PH and IPAH, involving genes associated with PAH, as well as those related to extracellular matrix (ECM) regulation and inflammation.

### 3.1. Genetic Variant Profile in COPD-PH

Pulmonary hypertension in COPD was associated with a trend toward greater genetic variability, particularly in severe PH, as compared with COPD patients without PH. Although no pathogenic variants were identified, some patients harboured VUS in genes that have been associated with PAH [9]. A patient with COPD-PH carried a novel VUS for this subpopulation in *BMPR2*, a gene definitively associated with PAH; however, two of the three computational predictors used in our analysis generally argue against a deleterious interpretation. One other patient harboured a VUS with a high CADD score in *BMPR1B* [10], which has disputed evidence for PAH association. The fact that, in COPD, VUS in PAH-associated genes were exclusive to patients with severe PH, with the so-called pulmonary vascular phenotype [3], suggests that PAH-associated genes may also contribute to the development of severe PH in COPD. Alternatively, these findings raise the possibility that patients carrying these VUS may actually have genetically driven IPAH with COPD as a comorbidity. Our observations further suggest that genetic testing could have clinical utility in this selected subgroup of patients.

In severe COPD-PH, we also identified VUS in genes not currently associated with PAH [8] that were absent in patients without PH and have plausible associations with PH. Two patients carried a VUS in *THBS1* (rs377693076), a gene reported in two studies of familial PAH [11,12] and involved in ECM regulation, angiogenesis, vascular remodelling, inflammation, and TGF-β signalling [13]. Two patients carried a VUS in *LOXL2* (rs759510676), which is upregulated in the serum of PH patients, experimental PH models, and IPAH lung tissue [14,15,16], and contributes to ECM deposition, potentially promoting vascular remodelling. Indeed, variant-driven LOXL2 dysregulation is consistent with the increased ECM protein expression shown in patients with severe COPD-PH [17,18]. However, our observations are based solely on computational predictions, and both variants had low-risk prediction scores. Therefore, these findings alone are insufficient to establish the functional significance of these VUS.

The increased variant burden in patients with COPD-PH involved genes related to mucin. Notably, variants in genes associated with MUC5 expression were highly prevalent among COPD-PH patients. The proportion of patients harbouring variants in *MUC5B* was higher in those with PH than in those without PH, while the number of variants per patient in *MUC5AC* was higher in severe COPD-PH than in nonsevere PH. In COPD, MUC5AC and MUC5B show opposing regulatory patterns, with MUC5AC upregulated in response to cigarette smoke and airway inflammation [19], while MUC5B tends to decline and is associated with preserved mucociliary function [20]. Although no association has been established between MUC5 gene variants and pulmonary vascular disease, our findings suggest that COPD patients with more severe PH may exhibit a heightened inflammatory state, reflected in greater genetic variability within the *MUC5AC* gene.

Overall, these findings suggest a genetic predisposition in a subset of patients with COPD-associated PH, particularly among those with severe PH. In fact, five out of 28 patients in this subgroup carried VUS in the tested genes. The variant profile in severe COPD-PH involved genes associated with PAH, as well as genes contributing to ECM regulation, consistent with the proteomic profile observed in this same cohort of patients [17].

### 3.2. Genetic Variant Profile in ILD-PH

In contrast to COPD-PH, patients with ILD-associated PH had a lower variant burden than those without PH. Only one patient with ILD-PH harboured a VUS in a gene with definitive evidence of association with PAH (*SMAD9*) [8]. Regarding genes not currently associated with PAH [9], two patients with ILD-PH carried a VUS in *TNFAIP6*, which has been linked to PH in systemic sclerosis and in IPAH [21,22]. However, the population frequency of this variant argues against a pathogenic role in PH; therefore, it should be regarded as an exploratory association, the biological relevance of which remains uncertain.

### 3.3. Genetic Divergence Between COPD-PH, ILD-PH and IPAH

Comparison of genetic profiles between CLD-PH and IPAH revealed both shared and distinct variant patterns. Compared with COPD-PH, patients with IPAH more frequently carried variants in *ACVRL1*, a gene with definitive PAH association, as well as in *SMAD3 (*also involved in the BMP/TGF-β pathway), *BCL2*, and *CYP1B1*, suggesting a greater contribution of genetic susceptibility in IPAH. Pathogenic and likely pathogenic variants in genes with definitive PAH association—*BMPR2* and *ACVRL1*—were exclusive to IPAH, while one patient with COPD-PH and one with ILD-PH harboured VUS in *BMPR2* and *SMAD9*, respectively.

Regarding VUS in genes with disputed evidence for PAH association, or in genes not currently considered PAH-associated [8], several patterns emerged. First, the variant rs1268550390 identified in *BMPR1B* in a patient with severe COPD-PH may be of interest, as it occurs in a gene with disputed PAH association, was exclusively observed in this specific phenotype, and was identified in a well-characterised patient cohort [23]. Second, in COPD, variants were identified exclusively in patients with severe PH, whereas no association with PH severity was observed in ILD-PH. Third, variant burden was higher in severe COPD-PH and IPAH, with six of 28 (21.4%) and 11 of 38 (28.9%) patients harbouring variants, respectively, compared with ILD-PH (9.3%). Fourth, among the three VUS identified in patients with CLD-PH, none was shared between COPD-PH and ILD-PH.

VUS in genes involved in ECM regulation were exclusive to severe COPD-PH, consistent with previous work identifying matrix remodelling as a key disturbed pathway in this subgroup of patients [17,24]. VUS in genes involved in the inflammatory response were detected in the ILD-PH and IPAH groups. Specifically, VUS in *PTGS2*, which is also involved in the prostacyclin pathway, were observed exclusively in IPAH, whereas VUS in *TNFAIP6* were exclusive to ILD-PH patients.

In addition, the VUS rs79204362 in *CYP1B1* was identified exclusively in three patients with IPAH. This variant had one of the highest CADD scores in the dataset (CADD = 30) and shows compelling biological plausibility for involvement in PAH, warranting further investigation. *CYP1B1* plays a key role in oestrogen metabolism and has been implicated in the female predominance of PAH [25], with reports linking genetic variation in this gene to the right ventricular function in women [26].

Overall, our results show partial genetic overlap between CLD-PH and IPAH, with genes involved in the BMP/TGF-β signalling axis and related pathways also identified in CLD-PH. However, disruption of this pathway appears to occur at different levels than in IPAH, as distinct genes within the pathway are affected in CLD-PH. Furthermore, we identified novel VUS with high predicted pathogenicity in genes not currently associated with PAH in patients with IPAH.

This study has limitations. First, the number of patients in each subgroup was limited due to the inclusion of only patients who underwent right heart catheterisation, the consideration of two different lung diseases, and stratification into three hemodynamic severity groups. Second, genetic variability assessment was based on a targeted panel containing 150 genes to focus on a sizeable number of genes that could be relevant in the pathogenesis of PH in both lung diseases. Accordingly, our findings are limited to these genes included in the panel. We cannot exclude the possibility that whole-exome or whole-genome sequencing would have identified additional variants, including variants in long non-coding genomic regions, such as long non-coding RNAs ([27]), that may be relevant to PH pathogenesis in lung disease. Further research using more comprehensive genomic approaches is warranted. Third, as copy number variant analysis was not performed, pathogenic exonic deletions or duplications cannot be excluded. And fourth, not all the genes currently considered with definitive evidence of PAH association were analysed. This was also due to the methodology adopted for panel design that focused on genes likely to influence PH development in CLD according to preestablished criteria, as described in Methods, based on the information available at the time of panel design.

## 4. Materials and Methods

### 4.1. Study Design and Patient Population

We included 63 patients with COPD and 51 with ILD, stratified according to hemodynamic severity assessed by means of right heart catheterisation (RHC), into three groups: no PH (control group; n = 17 with COPD and n = 19 with ILD), nonsevere PH (18 with COPD and 15 with ILD), and severe PH (28 with COPD and 17 with ILD). We also included 38 patients diagnosed with sporadic IPAH as a comparator group.

All patients had been referred for right heart catheterisation to our unit. Pre-capillary PH was defined as a mean pulmonary artery pressure >20 mmHg with a pulmonary artery wedge pressure ≤15 mmHg, and severe PH as a pulmonary vascular resistance (PVR) >5 Wood units, following current guidelines [2]. Patients with associated forms of PAH, other known causes of PH, or clinically significant cardiorespiratory comorbidities beyond COPD or ILD were excluded.

All patients underwent complete pulmonary function testing. COPD was diagnosed according to GOLD guidelines [28] in current or former smokers with a post-bronchodilator forced expiratory volume in 1 s/forced vital capacity (FEV_1_/FVC) ratio <0.7. For the present study, only patients with an FEV_1_ <80% of predicted were included. ILD diagnosis was confirmed through multidisciplinary discussion based on clinical characteristics, high-resolution CT scan, bronchoalveolar lavage, and lung biopsy patterns if appropriate [29,30]. The ILD group was restricted to patients with fibrosing idiopathic interstitial pneumonias or fibrosing chronic hypersensitivity pneumonitis. ILD associated with connective tissue disease, sarcoidosis, Langerhans’ cell histiocytosis, or combined pulmonary fibrosis and emphysema was excluded.

The study was approved by the Ethics Committee of Hospital Clínic (RE 2018/0709), and all participants provided written informed consent.

### 4.2. Gene Panel Design

A targeted gene panel was designed to capture genes potentially involved in the development and pathobiology of PH in COPD and ILD. A literature search was performed using PubMed (NCBI), DisGenet [31] and Ensembl Variation [32] databases. Disease-specific Medical Subject Headings (MeSHs) terms (chronic obstructive pulmonary disease, idiopathic pulmonary fibrosis, pulmonary hypertension) were used to curate relevant genes. Gene selection was conducted using a scoring system for PH, COPD, and ILD using the GUILD (Genes Underlying Inheritance Linked Disorders) [33] and DIAMOND (Disease Module Detection algorithm) [34] prioritisation algorithms. These scores reflected the network connectivity of each gene to disease-associated seed genes derived from genome-wide association studies, linkage analysis, and other curated sources. An integrated relevance score was then calculated by weighting conditions at 40% for PH and 30% each for COPD and ILD, reflecting the primary focus on PH. Top-ranked genes underwent manual curation based on biological plausibility and published evidence. A total of 150 genes were selected for inclusion (Supplementary Table S1, Figure S1). Among these, seven genes have definitive evidence and six have disputed evidence for a PAH gene–disease relationship [8]. The full interactome of selected genes is shown in Supplementary Figure S2.

### 4.3. Next Generation Sequencing (NGS)

Genomic DNA was extracted from frozen whole-blood samples. Next Generation Sequencing was conducted at the Functional Genomics Core Facility at IDIBAPS. Library preparation was performed with 200 ng of DNA. The xGen Library Prep MC kit with Unique Dual Indexes (UDIs) adapters was used, and probes targeting the coding regions of the selected genes (excluding UTRs) were synthesised by Integrated DNA Technologies (IDTs, United States, Iowa). Target enrichment was carried out by hybridisation capture. Libraries were sequenced on an Illumina NextSeq2000 platform with paired-end reads (2 × 151 bp). All samples achieved a minimum mean coverage >100×, with base quality scores (Q30) exceeding 90%.

### 4.4. Variant Annotation, Functional Effect Prediction and Statistical Analysis

After filtering and trimming adapter sequences, cleaned reads were aligned to the reference genome GRCh37/hg19 using Burrows–Wheeler Alignment (BWA-MEM v0.7.12). Duplicate reads were marked with the MarkDuplicates tool from Picard (v2.20.2-8). Variants were identified using Mutect2 and VarDict, as implemented in bcbio-nextgen. To improve the reliability of the variant calls, we retained only variants detected by both callers, with a minimum read depth of 20 and a variant allele fraction of at least 5%. A panel of normals generated from 1000 Genomes samples was also used to help remove potential false-positive calls. Variant annotation was performed on variant call format (VCF) files using SnpEff [35], the BcBio-nextgen Python toolkit (v.0.2.6) [36], and the Ensembl Variant Effect Predictor (VEP; release 114) to predict their genomic location and potential impact on encoded proteins [37]. Regarding coverage, depth over target regions was calculated using mosdepth 0.3.3 from the original alignments of the 153 samples. Coverage for each gene was calculated as the length-weighted mean coverage across its target regions. A more extended methodology is provided in the Supplementary Materials.

Only non-synonymous variants with high or moderate predicted impact (including missense, in-frame indels, frameshift mutations, and start/stop codon alterations) were retained for analysis. Only single-nucleotide variants and small indels were evaluated, as copy number variant detection was not included in the analysis pipeline. For the identification of pathogenic variants or VUS, patients with COPD or ILD without PH were used as the reference group; therefore, variants present in these patients were excluded from the analysis.

Variant pathogenicity was predicted using Combined Annotation-Dependent Depletion (CADD) [38], ClinVar [39], Mobidetails [40], allele frequency (AF) from gnomAD [41], REVEL (Rare Exome Variant Ensemble Learner) [42] and AlphaMissense [43]. Only variants with a CADD Phred score >20—which correspond to the top 1% most deleterious variants—were retained, and only those classified as pathogenic, likely pathogenic or variant of uncertain significance (VUS) were considered. Variant classification followed the American College of Medical Genetics and Genomics (ACMG) criteria, based on information derived from these resources [44].

Group comparisons in Table 1 were performed using the Kruskal–Wallis test with Dunn’s post hoc multiple comparisons corrected for multiple testing. For each group, we calculated the mean number of genes with at least one variant per patient across the 150-gene panel. This metric was obtained by summing, for each gene, the number of patients carrying at least one variant (allowing a patient to be counted multiple times if variants occurred in multiple genes) and dividing by the total number of patients in the group. The number of variants relative to the number of patients was calculated by summing all variants identified across the 150 genes and dividing by the number of patients in each group, and was expressed as a median and interquartile range (IQR). Group comparisons of variant burden were performed using the Kruskal–Wallis test followed by both corrected and uncorrected Dunn’s post hoc test. These comparisons are referred to as variant burden analysis, reflecting a descriptive quantification of variant load. For gene-specific analyses, the proportion of patients with variants was compared between groups using Fisher’s exact test.

## 5. Conclusions

In conclusion, our study shows limited genetic overlap between COPD-PH and ILD-PH and suggests a potentially greater genetic contribution to PH in COPD, particularly in its severe form, where a subset of patients carries VUS in PAH-associated genes or genes involved in vascular remodelling. Overall, these findings support distinct underlying pathogenic mechanisms in the development of PH in these two conditions. We also identified potentially deleterious variants in genes not currently associated with PAH, which may contribute to the development of PH across different clinical settings. These findings are hypothesis-generating and require independent replication and, where relevant, functional or orthogonal validation. Although our findings do not support the routine use of gene panel testing in CLD-associated PH, the presence of VUS in PAH-associated genes in a subset of patients with severe COPD-PH raises the possibility that genetic testing may have clinical utility in selected cases, as it could help identify individuals with pathogenic mechanisms overlapping those of PAH, potentially informing disease classification and individualised management. This possibility warrants further investigation.

## Figures and Tables

**Table 1:** Clinical, hemodynamic and functional characteristics of the study subjects.

Group	COPD			ILD			IPAH (*n* = 38)
	No PH (*n* = 17)	Nonsevere PH (*n* = 18)	Severe PH (*n* = 28)	No PH (*n* = 19)	Nonsevere PH (*n* = 15)	Severe PH (*n* = 17)	
Age, years	65.0 (60, 71) ^†^	62.3 (57, 69) ^†^	67.9 (64, 74) ^†^	69.5 (64, 79) ^†^	77.3 (73, 79) ^†^	75.0 (68, 78) ^†^	52.5 (43, 61)
Male sex, n (%)	12 (70.6) ^†^	10 (55.6) ^†^	23 (82.1) ^†^	13 (68.4) ^†^	10 (66.7) ^†^	12 (70.6) ^†^	7 (18.4)
BMI, Kg/m^2^	26.8 (23, 29)	25 (23, 31)	25.4 (23, 32)	27.4 (23, 31)	26.5 (24, 30)	24.9 (23, 28)	26.1 (21, 32)
Pulmonary function							
FVC, % pred	83 (67, 96)	70 (57, 79)^†^	81 (67, 95)	58 (43, 69) ^†^	62 (52, 76) ^†^	63 (56, 74) ^†^	91 (85, 98)
FEV_1_, % pred	46 (28, 61) ^†^	30 (22, 47)^†^	59 (39, 72) ^†^	65 (51, 79) ^†^	74 (64, 80)	71 (60, 81)	88 (80, 94)
FEV_1_/FVC, %	42 (31, 54) ^†^	41 (29, 53)^†^	54 (41, 64) ^†^	83 (78, 90)	82 (78, 87)	78 (73, 87)	74 (71, 81)
DLco, % pred	43 (34, 54)	31 (25, 46)^†^	27 (22, 34) *^†^	37 (31, 44) ^†^	34 (29, 38) ^†^	28 (16, 29) ^†^	63 (54, 70)
P_a_O_2_, mmHg	70 (60, 78)^†^	69 (61, 76)^†^	53 (49, 59) *^#†^	68 (57, 87)	68 (55, 79) ^†^	57 (52, 68) ^†^	79 (70, 93)
Pulmonary hemodynamics							
mPAP, mmHg	19 (16, 20) ^†^	25 (22, 33) ^†^	39 (34, 47) *^#^	17 (14, 20) ^†^	24 (22, 27) ^†^	39 (31, 42) *^†^	49 (42, 61)
CO, L/min	5.1 (4.4, 5.8) ^†^	5.0 (3.8, 5.2)	4.3 (3.5, 5.2)	4.7 (3.9, 5.8) ^†^	4.3 (3.9, 4.8)	3.6 (3.2, 4.4)	3.8 (3, 4.5)
CI, L/min/m^2^	2.6 (2.4, 3.1) ^†^	2.6 (2.3, 2.8) ^†^	2.3 (1.9, 2.7)	2.6 (2.2, 3.1) ^†^	2.5 (2.2, 2.9)	2.3 (1.9, 2.4)	1.9 (1.7, 2.5)
PAWP, mmHg	8 (6, 9)	9 (8, 10)	8 (6, 9)	6 (3, 8)	8 (5, 10)	7 (5, 9)	8 (6, 10)
PVR, WU	2 (1.6, 2.3) ^†^	3.5 (3.1, 5.7) ^†^	6.8 (5.5, 10.2) *^#^	2.3 (1.4, 2.6) ^†^	3.9 (3.5, 4.2) ^†^	6.7 (5.8, 10.1) *^#^	12.5 (7.7, 15.9)

Data are expressed as median and interquartile range (25th–75th percentile) or percentage, as appropriate. * *p* < 0.05 vs. no PH; # *p* < 0.05 vs. non-severe PH; † *p* < 0.05 vs. IPAH. Group comparisons were performed using the Kruskal–Wallis test with Dunn’s post hoc multiple comparisons corrected for multiple testing. Abbreviations: COPD: chronic obstructive pulmonary disease; ILD: interstitial lung disease; PH: pulmonary hypertension; IPAH: idiopathic pulmonary arterial hypertension; BMI: body mass index; 6-MWT: distance covered in the six-minute walking test; NT-proBNP: N-terminal pro-brain natriuretic peptide; FVC: forced vital capacity; FEV1: forced expiratory volume in the first second; DLco: diffusing capacity for carbon monoxide; PaO2: partial pressure of arterial oxygen; mPAP: mean pulmonary artery pressure; CO: cardiac output; CI: cardiac index; PAWP: pulmonary artery wedge pressure; PVR: pulmonary vascular resistance; WU: Wood units.

**Table 2:** Genes with pathogenic variants or variants of uncertain significance (VUS).

Gene Group	Gene	COPD		ILD		IPAH
		Nonsevere PH	Severe PH	Nonsevere PH	Severe PH	
PAH-associated	*BMPR2*		**1**			**3**
	*ACVRL1*					**1**
	*SMAD9*			**1**		
	*BMPR1B* *		**1**			
Matrix remodelling	*THBS1*		**2**			
	*LOXL2*		**2**			
Inflammation	*PTGS2*					**2**
	*TNFAIP6*			**1**	**1**	
Oxidative stress	*CYP1B1*					**3**
Signal transduction	*NCOR2*					**2**

Pathogenic and likely pathogenic variants (brown), and variants of uncertain significance (VUS) (blue) identified in the different patient groups, with the number of patients carrying variants in each gene (colour darkness in accordance). * *BMPR1B* has disputed evidence of association with PAH, while *BMPR2*, *ACVRL1* and *SMAD9* have definitive evidence [8].

## Data Availability

All data analysed during the current study are included in this article. Variant data have been submitted to ClinVar and are available under accession numbers SCV007614686, SCV007614687, SCV007614688, SCV007614689, SCV007614690, SCV007614691 and SCV007614692.

## Supplementary Materials

The following supporting information can be downloaded at https://www.mdpi.com/article/10.3390/ijms27208992/s1.

## Abbreviations

The following abbreviations are used in this manuscript:

PH	Pulmonary hypertension
CLD	Chronic lung disease
COPD	Chronic obstructive pulmonary disease
ILD	Interstitial lung disease
IPAH	Idiopathic pulmonary arterial hypertension
GWAS	Genome-wide association studies
PAH	Pulmonary arterial hypertension
DLCO	Diffusing capacity for carbon monoxide
*MUC5B*	Mucin-5B
*MUC5AC*	Mucin-5AC
*ACVRL1*	Activin A receptor-like type-1
*SMAD3*	SMAD family member-3
*BCL2*	B-cell CLL/lymphoma 2
*CYP1B1*	Cytochrome P450 family-1 subfamily-B member-1
*SIRT3*	Sirtuin-3
*TLR4*	Toll-like receptor-4
*BMPR2*	Bone morphogenetic protein receptor type-2
VUS	Variant of uncertain significance
*SMAD9*	SMAD family member-9
*BMPR1B*	Bone morphogenetic protein receptor type-1B
*THBS1*	Thrombospondin-1
*LOXL2*	Lysyl oxidase-like 2
*PTGS2*	Prostaglandin-endoperoxide synthase-2
*TNFAIP6*	TNF-alpha-induced protein 6
*NCOR2*	Nuclear repressor co-receptor 2
RHC	Right heart catheterization
PVR	Pulmonary vascular resistance
FEV_1_/FVC	Forced expiratory volume in 1 s/forced vital capacity
MeSH	Medical Subject Headings
GUILD	Genes Underlying Inheritance Linked Disorders
DIAMOND	Disease Module Detection algorithm
NGS	Next generation sequencing
UDIs	Unique Dual Indexes
VCF	Variant call format
CADD	Combined Annotation-Dependent Depletion
AF	Allele frequency
REVEL	Rare Exome Variant Ensemble Learner
ACMG	American College of Medical Genetics and Genomics
