# Supplementary material for: Targeted DNA Sequencing Reveals Distinct Rare-Variant Profiles in Pulmonary Hypertension Associated with Chronic Lung Diseases
Source: Int J Mol Sci. 2026 Oct 10;27(20):8992. doi: 10.3390/ijms27208992 (PMC13704505; doi:10.3390/ijms27208992)
Supplement: Supplementary file 1 [file ijms-27-08992-s001.zip › Supplementary materials_Bosacoma.pdf]

SUPPLEMENTARY MATERIALS

Genetic variant profiling reveals distinct genetic signatures in pulmonary hypertension associated with chronic lung diseases

Adelaida Bosacoma, Agustín R. Garcia, Irene Madrigal, Juan-José Lozano, Julia Sidorova, Daniel Aguilar, Isabel Blanco, Ylenia Roger, Olga Tura-Ceide, Anna Sardiné-Rama, Clara Martín-Ontiyuelo, Diego A. Rodríguez-Chiaradía, Manuel López-Meseguer, Fernanda Hernandez-Gonzalez, Jesús Ribas, Xavier Pomares, Salud Santos, María Molina-Molina, Rita Fernández-Hernández, Victor I. Peinado, Joan A. Barberà

Supplementary Methods

Read Processing and Alignment

Sequences were processed with the bcbio-nextgen pipeline (v1.1.2a0-f2b09f81). TruSeq adapters and low-quality bases were removed with cutadapt 1.18, and read quality was evaluated with FastQC 0.11.7. Reads were aligned with BWA-MEM 0.7.17 against the human reference genome GRCh38/hg38, in the 1000 Genomes version, which includes alternative contigs. PCR duplicates were marked (without removal) with biobambam2 2.0.87 (bamsormadup), and base qualities were recalibrated. Local realignment around indels was not performed.

Capture Design

Enrichment was performed by hybridization capture. The design consists of 3,640 120-bp probes covering 436,800 bp of target sequence across the 150 genes in the panel.

Variant Calling

Variants were called with two independent algorithms, VarDict (vardict-java 1.5.8) and GATK Mutect2 (GATK 4.0.11.0), and combined with the bcbio consensus module (bcbio-variation-recall). Only variants detected by both algorithms were retained. With this intersection strategy, specificity was prioritized over sensitivity.

A minimum allele frequency of 5% and a minimum depth of 20× were required. In VarDict, the following filters were also applied: at least 2 reads with the variant; total depth ≥ 3; mean mapping quality ≥ 10; mean base quality ≥ 22.5; strand bias, read-position bias, signal-to-noise ratio (≥ 1.5), and paired read number (≤ 5.25) filters.

Mutect2 calls were filtered with FilterMutectCalls using default parameters. Only variants with PASS status in both algorithms were retained. 4,792 calls were obtained at 1,365 distinct positions across the 152 analysed samples. Median depth at called positions was 710× (interquartile range 361–1,195×).

Coverage and Callable Regions

Depth over target regions was calculated with mosdepth 0.3.3 from the original alignments of the 153 samples, in two passes: with all reads; with only reads of mapping quality ≥ 20.

Coverage of each gene was calculated as the mean of its target regions weighted by their length.

Callability (callable fraction) was defined as the percentage of target bases with depth ≥40× and mapping quality ≥20. The 40× threshold is the depth required to detect the minimum allele frequency demanded (5%) with the two supporting reads required for the calling filter.

To separate the effect of global sample depth from the intrinsic behaviour of each gene,

relative coverage was also calculated: gene coverage divided by the panel mean coverage in the same sample.

Comparison with a Germline Calling Algorithm

To evaluate the published variants against a diploid germline model, calling was repeated on the same alignments with bcftools 1.13 (`mpileup` and `call -m`, diploid multiallelic model). Mapping and base qualities ≥20 were required, and a maximum depth of 10,000 was set over target regions extended 50 bp on each side.

Both variant sets were normalized with bcftools norm: left-alignment of indels and decomposition of multiallelic variants. A variant was considered to match between the two sets when sample, chromosome, position, and reference and alternative alleles matched, within the target regions.

Statistical Analysis

Differences between groups in coverage, callable fraction, and relative coverage: Kruskal-Wallis test. Relationship between number of variants and coverage: Spearman correlation between the number of variants of each patient in a gene and their coverage in that gene. Multiple comparisons: p-values for the 150 genes were corrected using the Benjamini-Hochberg method. Analyses were performed with Python 3.8.10, SciPy 1.10.1, statsmodels 0.13.5, and pandas 2.0.3.

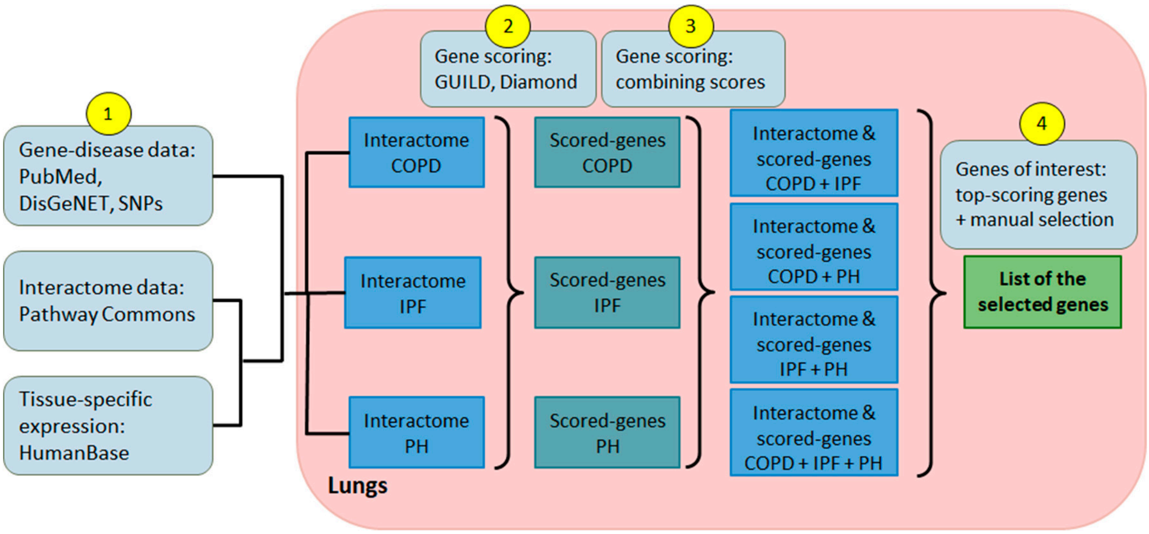

Supplementary Figure S1. Workflow diagram illustrating the methodology for gene panel construction. Numbered circles in yellow represent each step in the gene selection and prioritization process.

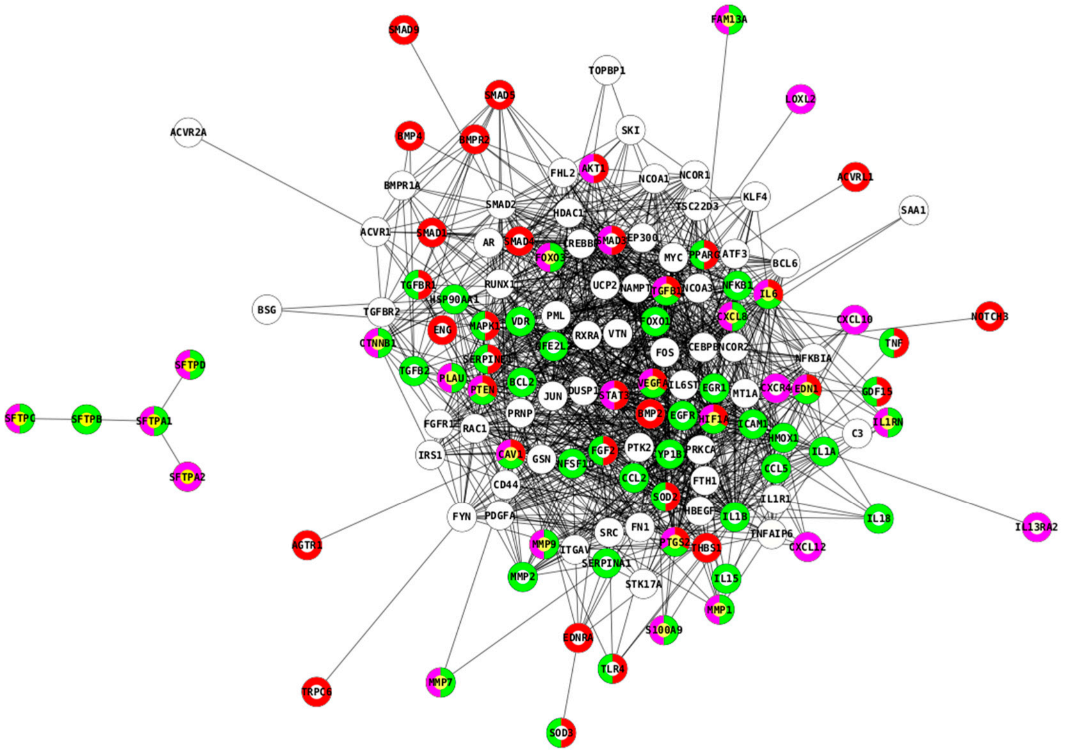

Supplementary Figure S2. Network of the final 150 genes visualized with Cytoscape. Condition-associated genes are marked with coloured borders: PH in red, COPD in green and ILD in pink. Nodes are shaded in yellow, with intensity corresponding to the composite score from GUILD and Diamond prioritization algorithm. The network reveals extensive interconnectivity among the genes, with central hubs such as *JUN*, *TGFB1*, *FOXO1* and *CCL2* showing a high number of connections (edges).

Supplementary Table S1. Final list of the 150 genes included in the targeted sequencing panel. For each gene, the table includes its disease association source, GUILD and Diamond scores for COPD, ILD and PH individually, as well as the combined score.

**Gene**	**COPD**	**ILD**	**PH**	**association to COPD**	**association to ILD**	**association to PH**	**association COPD · IPF · PH**	**association COPD · ILD**	**association COPD · PH**	**association ILD · PH**	**association COPD · ILD · PH**
**Score weights (%)**				0.3	0.3	0.4					
	Is source			Scores GUILD				Scores Diamond			
ACVR1				0.033	0.007	0.097	**0.000**				
ACVR2A				0.034	0.008	0.114	**0.000**				
ACVR2B				0.042	0.012	0.158	**0.000**				
ACVRL1			1	0.054	0.02	0.852	**0.001**				
AGER	1	1		0.536	0.507	0.074	**0.027**	1			
AGTR1			1	0.038	0.009	0.727	**0.000**				
AKT1		1	1	0.034	0.507	0.695	**0.016**	0.946	0.946	1	**0.946**
APCS				0.041	0.009	0.127	**0.000**				
APOA1				0.033	0.007	0.075	**0.000**				
AR				0.037	0.008	0.094	**0.000**	0.901	0.91	0.897	**0.857**
ATF3				0.038	0.008	0.096	**0.000**	0.874	0.888	0.915	**0.843**
BCL2	1			0.538	0.008	0.096	**0.001**	0.943	0.954	0.9	**0.9**
BCL6				0.036	0.007	0.09	**0.000**	0.887	0.886	0.915	**0.848**
BMP2			1	0.045	0.012	0.754	**0.001**	0.99	0.992	0.998	**0.99**
BMP4			1	0.041	0.014	0.794	**0.001**				
BMPR1A				0.033	0.007	0.096	**0.000**				
BMPR1B			1	0.065	0.03	1	**0.003**				
BMPR2			1	0.038	0.009	0.736	**0.000**				
BSG				0.03	0.005	0.065	**0.000**				
C3				0.045	0.011	0.128	**0.000**				
C4A				0.047	0.01	0.121	**0.000**				
CALCA	1			0.544	0.009	0.735	**0.005**		1		
CASP3				0.035	0.006	0.08	**0.000**	0.818	0.88	0.797	**0.757**
CAV1	1	1	1	0.541	0.51	0.735	**0.270**	1	1	1	**1**
CCL2	1			0.552	0.013	0.764	**0.007**	0.996	1	0.996	**0.996**
CCL5	1			0.559	0.015	0.167	**0.002**	0.989	0.988	0.977	**0.977**
CD44				0.04	0.009	0.113	**0.000**	0.865	0.879	0.858	**0.808**
CDKN1A	1			0.536	0.007	0.085	**0.000**	0.973	0.983	0.956	**0.956**
CEBPB				0.037	0.008	0.096	**0.000**	0.979	0.986	0.979	**0.972**
CREBBP				0.034	0.006	0.079	**0.000**	0.957	0.976	0.944	**0.939**
CTNNB1	1	1		0.533	0.506	0.075	**0.027**	1	0.663	0.663	**0.663**
CXCL10		1		0.056	0.514	0.158	**0.006**				
CXCL12		1		0.049	0.513	0.151	**0.005**				

CXCL13		1		0.105	0.529	0.217	**0.016**				
CXCL8	1	1		0.548	0.512	0.748	**0.280**	1	1	1	**1**
CXCR4		1		0.046	0.511	0.129	**0.004**	0.892	0.698	0.782	**0.698**
CYP1B1	1			0.547	0.011	0.133	**0.001**	0.994	0.985	0.979	**0.979**
DSP	1	1		0.534	0.507	0.066	**0.024**	1			
DUSP1				0.04	0.009	0.105	**0.000**	0.881	0.874	0.892	**0.829**
EDN1	1	1	1	0.549	0.513	0.764	**0.287**	1	1	1	**1**
EDNRA			1	0.053	0.012	0.774	**0.001**		0.658		
EGFR	1			0.536	0.007	0.087	**0.000**	0.946	0.942	0.891	**0.891**
EGR1	1			0.542	0.009	0.113	**0.001**	0.978	0.989	0.967	**0.967**
EIF2AK4		1	1	0.029	0.505	0.674	**0.013**			1	
ENG			1	0.046	0.013	0.777	**0.001**				
EP300				0.034	0.006	0.079	**0.000**	0.93	0.946	0.923	**0.901**
FAM13A	1	1		0.538	0.508	0.094	**0.034**	1			
FGF2	1		1	0.545	0.011	0.748	**0.006**	0.945	1	0.945	**0.945**
FGFR1				0.039	0.009	0.102	**0.000**	0.864	0.885	0.869	**0.815**
FHL2				0.037	0.008	0.1	**0.000**	0.87	0.905	0.92	**0.851**
FN1				0.036	0.008	0.096	**0.000**	0.882	0.937	0.893	**0.859**
FOS				0.04	0.009	0.109	**0.000**	0.977	0.975	0.974	**0.963**
FOXO1	1			0.538	0.008	0.713	**0.004**	0.964	1	0.964	**0.964**
FOXO3	1	1		0.535	0.507	0.085	**0.031**	1	0.911	0.911	**0.911**
FTH1				0.039	0.008	0.098	**0.000**	0.945	0.905	0.894	**0.874**
FYN				0.036	0.008	0.092	**0.000**	0.807	0.797	0.777	**0.707**
GDF15	1		1	0.547	0.011	0.747	**0.006**		1		
GSN				0.037	0.008	0.093	**0.000**	0.869	0.899	0.853	**0.816**
HBEGF				0.043	0.01	0.118	**0.000**	0.943	0.929	0.937	**0.906**
HDAC1				0.031	0.005	0.067	**0.000**	0.882	0.877	0.908	**0.838**
HIF1A	1	1	1	0.535	0.507	0.701	**0.254**	1	1	1	**1**
HSP90AA1	1			0.53	0.005	0.06	**0.000**	0.813	0.806	0.655	**0.655**
HTR2A	1		1	0.544	0.01	0.726	**0.005**		1		
ICAM1	1			0.544	0.01	0.12	**0.001**	0.993	0.998	0.991	**0.991**
IL13RA2		1		0.074	0.518	0.189	**0.010**				
IL15	1			0.553	0.013	0.139	**0.001**	0.894	0.839	0.75	**0.75**
IL18	1			0.553	0.012	0.145	**0.001**	0.893	0.837	0.747	**0.747**
IL1A	1			0.552	0.013	0.142	**0.001**	0.984	0.96	0.945	**0.945**
IL1B	1			0.549	0.012	0.136	**0.001**	0.999	0.999	0.998	**0.998**
IL1R1				0.05	0.012	0.131	**0.000**	0.971	0.876	0.866	**0.859**
IL1RN	1	1		0.55	0.512	0.135	**0.051**	1			
IL6	1	1	1	0.549	0.513	0.759	**0.285**	1	1	1	**1**
IL6ST				0.046	0.01	0.121	**0.000**	0.902	0.897	0.825	**0.817**
IRS1				0.039	0.009	0.104	**0.000**	0.843	0.812	0.79	**0.736**
ITGAV				0.041	0.01	0.118	**0.000**	0.883	0.904	0.859	**0.828**
JUN				0.034	0.007	0.084	**0.000**	0.967	0.968	0.959	**0.948**
KCNK3			1	0.032	0.006	0.682	**0.000**				

KLF4				0.042	0.009	0.112	**0.000**	0.955	0.959	0.927	**0.922**
LOXL2		1		0.048	0.513	0.16	**0.005**				
MAPK1	1		1	0.534	0.006	0.694	**0.003**	0.934	1	0.934	**0.934**
MMP1	1	1		0.548	0.511	0.138	**0.052**	1			
MMP2	1			0.545	0.011	0.134	**0.001**	0.918	0.984	0.903	**0.903**
MMP7	1	1		0.549	0.512	0.138	**0.052**	1			
MMP9	1	1		0.551	0.513	0.16	**0.060**	1	0.9	0.9	**0.9**
MT1A				0.047	0.011	0.135	**0.000**	0.932	0.941	0.9	**0.888**
MUC5AC	1	1		0.579	0.555	0.035	**0.015**	1			
MUC5B	1	1		0.541	0.512	0.116	**0.043**	1			
MYC				0.03	0.005	0.062	**0.000**	0.889	0.855	0.871	**0.814**
NAMPT				0.039	0.008	0.101	**0.000**	0.989	0.984	0.979	**0.976**
NCOA1				0.036	0.007	0.089	**0.000**	0.886	0.898	0.893	**0.843**
NCOA3				0.034	0.007	0.081	**0.000**	0.879	0.888	0.885	**0.831**
NCOR1				0.033	0.006	0.076	**0.000**	0.862	0.87	0.888	**0.816**
NCOR2				0.036	0.007	0.09	**0.000**	0.935	0.952	0.936	**0.913**
NFE2L2	1			0.537	0.007	0.09	**0.000**	0.972	0.975	0.948	**0.948**
NFKB1	1			0.536	0.007	0.084	**0.000**	0.985	0.992	0.977	**0.977**
NFKBIA				0.037	0.007	0.087	**0.000**	0.981	0.97	0.954	**0.953**
NOS3	1		1	0.531	0.006	0.69	**0.003**		1		
NOTCH3			1	0.04	0.009	0.725	**0.000**				
PDGFA				0.046	0.011	0.14	**0.000**	0.904	0.975	0.919	**0.9**
PLAU	1	1		0.543	0.51	0.119	**0.044**	1	0.913	0.913	**0.913**
PML				0.037	0.007	0.09	**0.000**	0.979	0.986	0.973	**0.969**
PPARG	1		1	0.541	0.009	0.726	**0.005**	0.958	1	0.958	**0.958**
PRKCA				0.038	0.008	0.102	**0.000**	0.937	0.961	0.965	**0.932**
PRNP				0.037	0.007	0.092	**0.000**	0.938	0.932	0.933	**0.903**
PTEN	1	1	1	0.534	0.507	0.697	**0.252**	1	1	1	**1**
PTGS2	1	1	1	0.549	0.512	0.761	**0.285**	1	1	1	**1**
PTK2				0.037	0.008	0.096	**0.000**	0.898	0.904	0.872	**0.842**
RAC1				0.035	0.007	0.085	**0.000**	0.876	0.886	0.857	**0.815**
RUNX1				0.037	0.008	0.1	**0.000**	0.884	0.907	0.935	**0.866**
RXRA				0.036	0.007	0.091	**0.000**	0.876	0.885	0.883	**0.827**
S100A9	1	1		0.542	0.509	0.113	**0.042**	1			
SAA1				0.052	0.012	0.136	**0.000**				
SERPINA1	1			0.543	0.01	0.118	**0.001**				
SERPINE1	1		1	0.544	0.01	0.747	**0.005**	0.99	1	0.99	**0.99**
SFTPA1	1	1		0.952	1	0	**0.000**	1			
SFTPA2		1		0.494	0.92	0	**0.000**				
SFTPB	1			0.963	0.479	0	**0.000**				
SFTPC	1	1		1	0.902	0	**0.000**	1			
SFTPD	1	1		0.986	0.92	0	**0.000**	1			
SIRT3				0.031	0.006	0.067	**0.000**				
SKI				0.035	0.008	0.101	**0.000**				

SLC6A4	1		1	0.558	0.018	0.866	**0.012**		1		
SMAD1			1	0.033	0.007	0.701	**0.000**				
SMAD2				0.032	0.006	0.076	**0.000**	0.877	0.888	0.889	**0.832**
SMAD3		1	1	0.036	0.508	0.712	**0.017**	0.942	0.942	1	**0.942**
SMAD4			1	0.033	0.007	0.702	**0.000**	0.865	0.92	0.94	**0.865**
SMAD5			1	0.032	0.006	0.701	**0.000**				
SMAD9			1	0.033	0.007	0.773	**0.000**				
SOD2	1		1	0.539	0.008	0.715	**0.004**	0.995	1	0.995	**0.995**
SOD3	1		1	0.563	0.016	0.86	**0.010**		1		
SRC				0.037	0.008	0.089	**0.000**	0.757	0.758	0.74	**0.652**
STAT3		1	1	0.037	0.508	0.707	**0.018**	0.998	0.998	1	**0.998**
STK17A				0.047	0.011	0.133	**0.000**	0.94	0.926	0.939	**0.904**
STN1	1	1		0.53	0.506	0.061	**0.022**	1			
TERT	1	1		0.532	0.504	0.056	**0.020**	1			
TGFB1	1	1	1	0.539	0.509	0.725	**0.265**	1	1	1	**1**
TGFB2	1			0.546	0.013	0.17	**0.002**		0.685		
TGFBR1	1		1	0.536	0.008	0.721	**0.004**		1		
TGFBR2				0.041	0.01	0.127	**0.000**	0.675	0.791	0.688	**0.606**
THBS1			1	0.045	0.011	0.758	**0.001**	0.634	0.897	0.707	**0.634**
TLR4	1		1	0.549	0.011	0.75	**0.006**		1		
TNF	1		1	0.55	0.012	0.759	**0.007**		1		
TNFAIP6				0.06	0.017	0.2	**0.000**	0.905	0.905	0.994	**0.903**
TNFSF10	1			0.547	0.011	0.126	**0.001**	0.981	0.991	0.972	**0.972**
TOPBP1				0.029	0.004	0.054	**0.000**				
TRPC6			1	0.039	0.009	0.72	**0.000**				
TSC22D3				0.039	0.008	0.103	**0.000**	0.888	0.882	0.922	**0.85**
UCP2				0.039	0.008	0.102	**0.000**	0.902	0.94	0.897	**0.872**
VEGFA	1	1	1	0.54	0.509	0.724	**0.265**	1	1	1	**1**
VTN				0.053	0.015	0.18	**0.000**				

List of the genes with their scores. Source: their association with the disease is described. Scores according to the GUILD and Diamond methodologies for each condition (COPD, ILD, PH) and for all three together.

Supplementary Table S2. List of patients with pathogenic, likely pathogenic variants or variants of uncertain significance in patients with pulmonary hypertension[1].

Patient #	Patient group	Sex	Age	mPAP (mmHg)	PVR (WU)	Gene	#Uploaded variation	Chromosome location	MANE SELECT	Consequence	Allele change	Amino acid change	ClinVar/ Mobidetails	CADD	Allele Frequency	REVEL	Alpha Missense
1	COPD-SPH	M	70	42	8.3	*BMPR2*	rs573463511	Chr2:203332274	NM_001204.7	Missense	c.80C>T	p.(Ser27Leu)	B/VUS	22.8	1.9x10^{-5}	0.311	0.1552
2	COPD-SPH	M	66	35	5.5	*BMPR1B**	rs1268550390	Chr4:96052366	NM_001203.3	Missense, splice region	c.779G>T	p.(Gly260Val)	VUS	34	4x10^{-6}	0.888	0.9286
						LOXL2	rs759510676	Chr8:23167289	NM_002318.3	Missense	c.1772C>A	p.(Thr591Lys)	VUS	22.5	1.7x10^{-5}	0.086	0.0907
3	COPD-SPH	M	68	33	6.6	*LOXL2*	rs759510676	Chr8:23167289	NM_002318.3	Missense	c.1772C>A	p.(Thr591Lys)	VUS	22.5	1.7x10^{-5}	0.086	0.0907
4	COPD-SPH	M	66	58	18.7	*THBS1*	rs377693076	Chr15:39882031	NM_003246.4	Missense	c.1952C>T	p.(Thr651Met)	B/VUS	22.3	5.4x10^{-4}	0.229	0.0764
5	COPD-SPH	M	70	50	13.7	*THBS1*	rs377693076	Chr15:39882031	NM_003246.4	Missense	c.1952C>T	p.(Thr651Met)	B/VUS	22.3	5.4x10^{-4}	0.229	0.0764
6	ILD-NSPH	M	78	26	3.7	*SMAD9*	rs941764904	Chr13:37453823	NM_001127217.3	Missense	c.4C>G	p.(His2Asp)	VUS	23.6	4x10^{-6}	0.524	0.1683
7	ILD-NSPH	M	77	27	4.8	*TNFAIP6*	rs75961064	Chr2:152220510	NM_007115.4	Missense	c.148A>G	p.(Thr50Ala)	B/VUS	26.2	1.5x10^{-2}	0.515	0.4524
8	ILD-SPH	M	82	42	9.3	*TNFAIP6*	rs75961064	Chr2:152220510	NM_007115.4	Missense	c.148A>G	p.(Thr50Ala)	B/VUS	26.2	1.5x10^{-2}	0.515	0.4524
9	IPAH	F	46	57	18	*BMPR2*	rs1085307203	Chr2:203332274	NM_001204.7	Missense	c.280T>C	p.(Cys94Arg)	P	28.9	-	0.953	0.9962
10	IPAH	F	26	42	16.8	*BMPR2*	rs137852744	Chr2:203332274	NM_001204.7	Missense	c.1040G>A	p.(Cys347Tyr)	P	31	-	0.939	0.9936
11	IPAH	M	18	76	17.5	*BMPR2*	rs137852748	Chr2:203332274	NM_001204.7	Stop gained	c.2617C>T	p.(Arg873*)	P	42	-	-	-
12	IPAH	M	70	49	12.6	*ACVRL1*	rs1234976168	Chr12:52309927	NM_000020.3	Missense	c.1156C>T	p.(Arg386Cys)	LP	29.9	-	0.788	0.1807
13	IPAH	M	60	27	4.3	*CYP1B1*	rs79204362	Chr2:38298394	NM_000104.4	Missense	c.1103G>T	p.(Arg368Leu)	B/VUS	30	3.1x10^{-3}	-	0.2003
14	IPAH	F	63	25	3.8	*CYP1B1*	rs79204362	Chr2:38298394	NM_000104.4	Missense	c.1103G>T	p.(Arg368Leu)	B/VUS	30	3.1x10^{-3}	-	0.2003
15	IPAH	F	50	40	6	*CYP1B1*	rs79204362	Chr2:38298394	NM_000104.4	Missense	c.1103G>T	p.(Arg368Leu)	B/VUS	30	3.1x10^{-3}	-	0.2003
16	IPAH	F	38	36	9	*NCOR2*	rs200297509	Chr12:124835279	NM_006312.6	Missense	c.3698G>A	p.(Thr1233Met)	LB/VUS	27.9	8.6x10^{-4}	0.605	0.6539
17	IPAH	F	52	44	7.3	*NCOR2*	rs200297509	Chr12:124835279	NM_006312.6	Missense	c.3698G>A	p.(Thr1233Met)	LB/VUS	27.9	8.6x10^{-4}	0.605	0.6539
18	IPAH	F	33	66	23	*PTGS2*	rs5273	Chr1:186643768	NM_000963.4	Missense	c.1532T>G	p.(Val511Ala)	B/VUS	20.4	4.4x10^{-3}	0.163	0.2664
19	IPAH	F	46	51	15	*PTGS2*	rs5273	Chr1:186643768	NM_000963.4	Missense	c.1532T>G	p.(Val511Ala)	B/VUS	20.4	4.4x10^{-3}	0.163	0.2664

[1]Variants absent in patients without pulmonary hypertension.

For each patient, the variants that they carry, with the gene, variant ID, chromosomal coordinate, cDNA position, predicted consequence, amino acid change, and pathogenicity predictions from CADD, allele frequency, REVEL and AlphaMissense are provided. Only variants with a CADD Phred score >20 are included. *All genes have definitive evidence except for BMPR1B, with disputed evidence [8]. Abbreviations: mPAP: mean pulmonary artery pressure; PVR: pulmonary vascular resistance; WU: Wood units; SPH: severe pulmonary hypertension; NSPH: nonsevere pulmonary hypertension; F: female; M: male; CADD: Combined Annotation-Dependent Depletion; REVEL: Rare Exome Variant Ensemble Learner; P: pathogenic; LP: likely pathogenic; B: benign; VUS: variant of uncertain significance.
